# Intravenous Clarithromycin in Critically Ill Adults: A Population Pharmacokinetic Study

**DOI:** 10.3390/antibiotics14060559

**Published:** 2025-05-30

**Authors:** Reya V. Shah, Karin Kipper, Emma H. Baker, Charlotte I. S. Barker, Isobel Oldfield, Harriet C. Davidson, Cleodie C. Swire, Barbara J. Philips, Atholl Johnston, Andrew Rhodes, Mike Sharland, Joseph F. Standing, Dagan O. Lonsdale

**Affiliations:** 1 Institute for Infection and Immunity, City St George’s, University of London, London SW17 0RE, UK; 2 Department of Clinical Pharmacology & Therapeutics, St George’s University Hospitals NHS Foundation Trust, London SW17 0QT, UK; 3 Institute of Chemistry, University of Tartu, 50411 Tartu, Estonia; 4 Analytical Services International Ltd., London SW17 0RE, UK; 5 Department of Medical and Molecular Genetics, King’s College London, London WC2R 2LS, UK; 6 UCL Great Ormond Street Institute of Child Health, London WC1N 1EH, UK; 7 Infection Care Group, St George’s University Hospitals NHS Foundation Trust, London SW17 0QT, UK; 8 Brighton and Sussex Medical School, Brighton BN1 9PX, UK; 9 Clinical Pharmacology, William Harvey Research Institute, Queen Mary University of London, London WC1E 7HU, UK; 10 Department of Critical Care, St George’s University Hospitals NHS Foundation Trust, London SW17 0QT, UK; 11 Great Ormond Street Hospital for Children NHS Foundation Trust, London WC1N 3JH, UK

**Keywords:** pharmacometrics, clarithromycin, critical illness, macrolides

## Abstract

**Background:** Clarithromycin is a commonly used macrolide antibiotic. Infection is a major source of mortality and morbidity in critical care units. Pharmacokinetics may vary during critical illness and suboptimal antimicrobial exposure has been shown to be associated with treatment failure. The pharmacokinetics of intravenous clarithromycin in critical illness have not previously been described. **Methods:** Pharmacokinetic, clinical and demographic data were collected from critically ill adults receiving intravenous clarithromycin. Drug concentrations were measured using high-performance liquid chromatography/mass spectrometry. Population pharmacokinetic analysis was performed using NONMEM version 7.5.1. Allometric weight scaling was added, and periods of renal replacement therapy were excluded a priori. Simulations of 10,000 patients were performed to assess pharmacokinetic–pharmacodynamic (PKPD) target attainment. **Results:** The analysis included 121 samples taken from 19 participants. A two-compartment model was found to provide the best fit. The addition of covariates did not improve model fit. There was no evidence of auto-inhibition in this population. Population parameter estimates of clearance and volume of distribution were lower than previously reported, with high interindividual variability. Simulations suggested reasonable pharmacokinetic–pharmacodynamic (PKPD) target attainment with current dosing regimens for most organisms that clarithromycin is used to treat with known clinical breakpoints. **Conclusions:** To our knowledge, this is the first study to describe the pharmacokinetics of intravenous clarithromycin in humans. Although our simulations suggest reasonable target attainment, further investigation into appropriate PKPD targets and clinical breakpoints for clarithromycin may enable dosing optimisation in this population.

## 1. Introduction

Clarithromycin is a semi-synthetic macrolide with a broad-spectrum of antimicrobial activity [1]. The impact of critical illness and associated organ dysfunction on clarithromycin pharmacokinetics has not been described, and dosing recommendations are identical in critically and non-critically ill populations. The pharmacokinetics of many antibiotics have been found to be highly variable during critical illness [2]. Suboptimal pharmacokinetic exposure of antimicrobials has been shown to be associated with treatment failure [3,4], and optimising exposure to antibiotics, most notably beta-lactams, has demonstrated improved clinical outcomes in critically ill patients who have an infection [4].

The pharmacokinetics of oral clarithromycin in clinically well individuals have been described [5,6,7]. Intravenous clarithromycin pharmacokinetics have only previously been studied in foals [8]. The only published study in a human critical care setting studied enteral administration of clarithromycin via a nasogastric tube. In their study, Fish and Abraham found adequate absorption and results comparable to those found in studies of healthy volunteers or less seriously ill patients [9]. The study excluded patients with organ dysfunction (specifically renal, hepatic and gastrointestinal dysfunction). We aimed to address this knowledge gap with a pharmacokinetic study of clarithromycin in critically ill adults.

Macrolide antibiotics exert bacteriostatic action through inhibition of protein synthesis. Clarithromycin displays broad spectrum activity against both Gram-negative and Gram-positive organisms and mycobacteria. It is commonly used to treat infections of the respiratory tract, skin and pharynx. Clarithromycin is usually used to cover atypical infection or as an alternative agent in penicillin allergy. Clarithromycin is often used via enteral administration. The intravenous route may be used if the enteral route is unavailable, and intravenous administration may be used preferentially in severe illness or unreliable enteral absorption. The major metabolite of clarithromycin, 14-OH-clarithromycin, also possesses potent antibiotic activity [5].

Macrolides display concentration- and time-dependent activity and various pharmacokinetic–pharmacodynamic (PKPD) targets have been associated with efficacy. For example, Kays and Denys reported the fraction of time above the mean inhibitory concentration (MIC) as a measure of clarithromycin efficacy against clinical *Streptococcus pneumoniae* isolates [10], and Novelli et al. (2002) found the ratio of the peak concentration compared to the MIC (C_max_:MIC) to be the best predictor of successful clarithromycin treatment in a murine thigh infection and peritonitis model [11].

Tessier et al. (2002), tested a separate murine model of pneumococcal pneumonia, testing various PKPD indices [12]. This study suggested that the ratio of exposure in 24 h compared to the MIC (free 24 h AUC:MIC), which accounts for both time and concentration, was the PKPD index that best predicted the activity of clarithromycin, although correlation was similar for C_max_:MIC. This study demonstrated that a total 24 h AUC:MIC for total clarithromycin (not accounting for protein binding) of greater than 100 was associated with bactericidal activity and positive outcomes [12]. Clarithromycin is approximately 80% bound to plasma proteins at therapeutic levels [13], although binding reduces with increasing concentrations [5]. Clinical targets have been defined as a free 24 h AUC:MIC of 25–35 [14], with some studies requiring a more conservative target of a free 24 h AUC:MIC of at least 100 [15].

Common organisms associated with pneumonia that may be treated by clarithromycin have differing clinical breakpoints for macrolides according to the European Committee on Antimicrobial Susceptibility Testing (EUCAST). *Streptococcus pneumoniae*, Streptococcal groups A, B, C and G and *Moraxella catarrhalis* all have a sensitive and resistant breakpoint of 0.25 mg/L to macrolide antibiotics. For *Staphylococcus* spp. (including *S. aureus*), the sensitive and resistant breakpoint is 1 mg/L [16]. The EUCAST notes that clinical evidence for macrolide efficacy against *Haemophilus influenzae* is conflicting, due to high spontaneous cure rates, but recommends the use of the epidemiological cut-off (ECOFF) of 32 mg/L if testing is required. *Legionella pneumophila* is an important cause of pneumonia and may cause critical illness. However, the EUCAST notes that there is no established reference method, nor any documentation of clinical outcomes related to antimicrobial susceptibility testing, with no clinical breakpoints available for this organism. *Chlamydia pneumoniae* is also an important cause of pneumonia for which there are no available breakpoints. The EUCAST does not have available breakpoints for *Mycoplasma pneumoniae*, which causes pneumonia, but the Clinical and Laboratory Standards Institute (CLSI) has published antimicrobial susceptibility testing guidance for human *Mycoplasma* spp., with a MIC above 1 mg/L considered resistant and below 0.5 mg/L considered sensitive for macrolides [17]. Of note, the incidence of macrolide-resistant *Mycoplasma pneumonia* is increasing globally, particularly in eastern Asia [18], and macrolide resistant strains usually have a MIC above 16 mg/L [17].

Clarithromycin inhibits CYP3A4 enzymes and may interact with co-administered drugs by reducing metabolism via this pathway and increasing exposure. It has also been shown to autoinhibit its own metabolism, particularly at higher doses [7]. Clarithromycin has also been shown to have an immunomodulatory effect [19].

Alongside describing the pharmacokinetics of clarithromycin, we aimed to explore the extent to which antimicrobial PKPD targets are met with current dosing recommendations and to explore whether autoinhibition impacts drug exposure in critically ill populations. To our knowledge, this is the first study describing intravenous clarithromycin pharmacokinetics in critically ill adults.

## 2. Results

### 2.1. Baseline Characteristics

During the study period, 139 samples were taken from 22 participants, of which 18 samples were excluded from 5 participants due to being taken during periods of renal replacement therapy. The analysis included 121 pharmacokinetic samples from 19 participants. Participant characteristics are shown in Table 1.

During the period of recruitment, 12 participants had periods requiring intubation and ventilation, with 14 having periods where they were spontaneously breathing, five patients required periods of non-invasive ventilation and 12 participants received vasopressor support during the study. During the study, 12 participants had at least one recording of a blood plasma pH below the normal physiological range of 7.35–7.45. Concomitant drugs were evaluated for interactions using the British National Formulary [20], and no drugs were predicted to impact clarithromycin pharmacokinetics. A list of concomitant medications is available in the Supplementary Materials.

**Figure 1:**
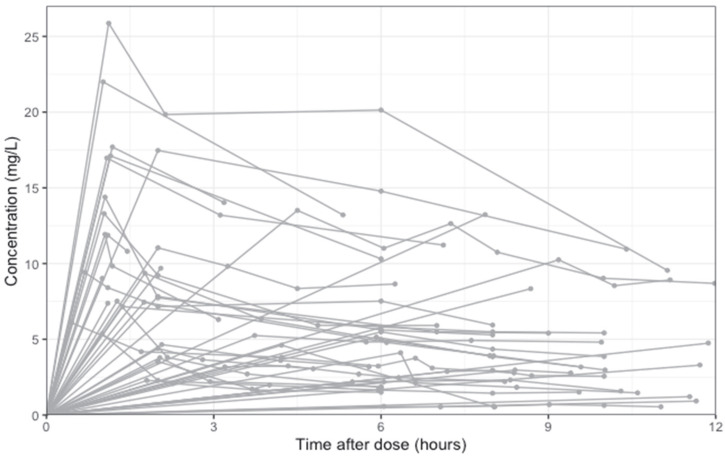
Clarithromycin concentration over time after administration. Crude concentration time curve with individual samples represented by a point. Individual dosing intervals for each participant (*n* = 19) are represented by a separate line. A concentration of 0 mg/L was simulated for the start of each dosing interval per participant. Not all participants had a measurement of peak concentrations and lines may not accurately represent their expected concentration profile.

Patients included in this analysis were receiving intravenous clarithromycin at a dose of 500 mg every 12 hours. The majority of participants contributed eight samples to the analysis, with four participants contributing four or fewer samples. Not all participants had peak concentrations measured. The raw data are shown by participant in Figure 1.

### 2.2. Pharmacokinetic Analysis

A two-compartment model was found to provide the best fit with parameter estimates shown in Table 2.

The addition of albumin, creatinine, the presence of liver disease, sex, height and age to parameters did not provide any significant improvement in the model fit. Interoccasional variability was tested but there was no evidence of auto-inhibition in this cohort of patients. Subsequent investigation of non-linear pharmacokinetics using a Michaelis–Menten elimination model did not improve the model fit for this cohort.

### 2.3. Evaluation Methods

**Figure 2:**
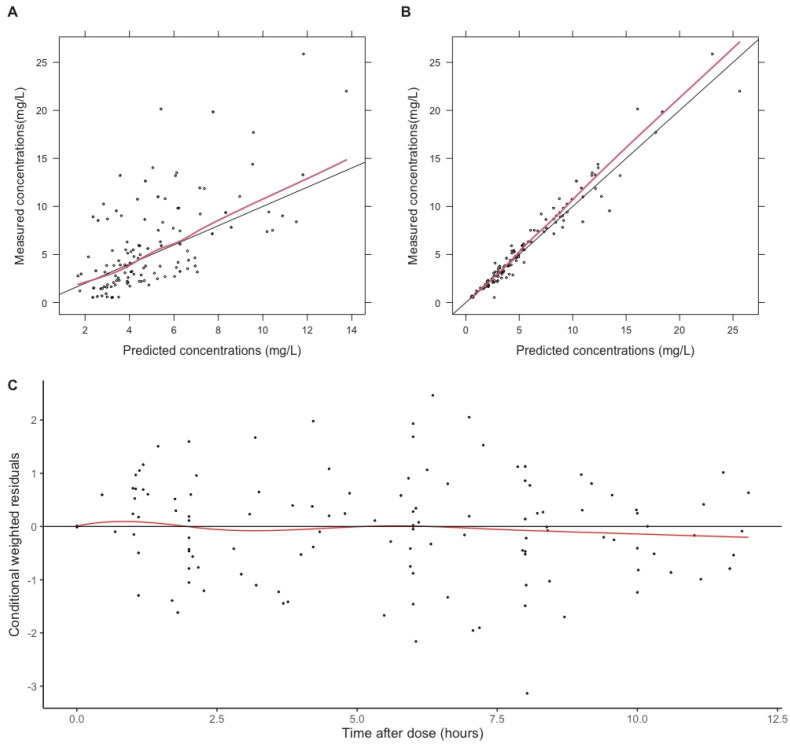
Goodness-of-fit plots. Goodness-of-fit plots for the final model. (**A**) Observed concentrations against population predictions; (**B**) observed concentrations against individual predictions; (**C**) conditional weighted residuals against time after dose. The red lines represent a smooth curve of the observed data. The black line represents the line of identity (plots (**A**,**B**)) and the zero line (plot (**C**)).

**Figure 3:**
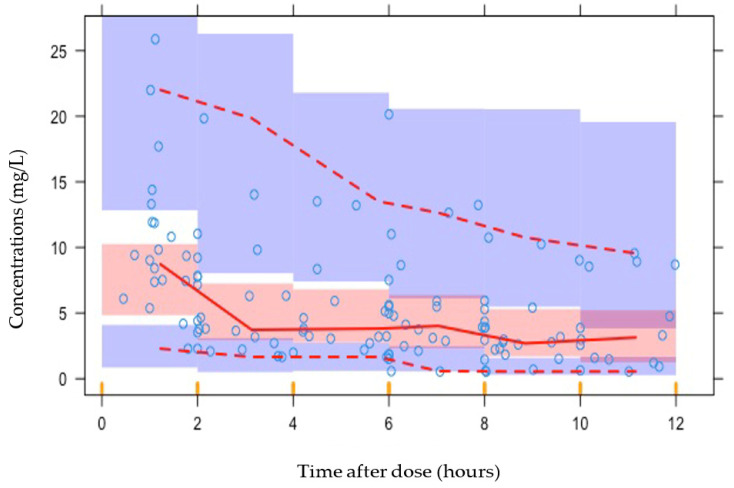
Visual predictive plot. The median is represented by the solid red line with the dashed red lines representing the 95% confidence intervals. The orange dashes along the x-axis represent the binning at specific time points.

Goodness-of-fit plots (Figure 2) and visual predictive curves (Figure 3) demonstrated a reasonable fit of data.

### 2.4. Simulations

**Figure 4:**
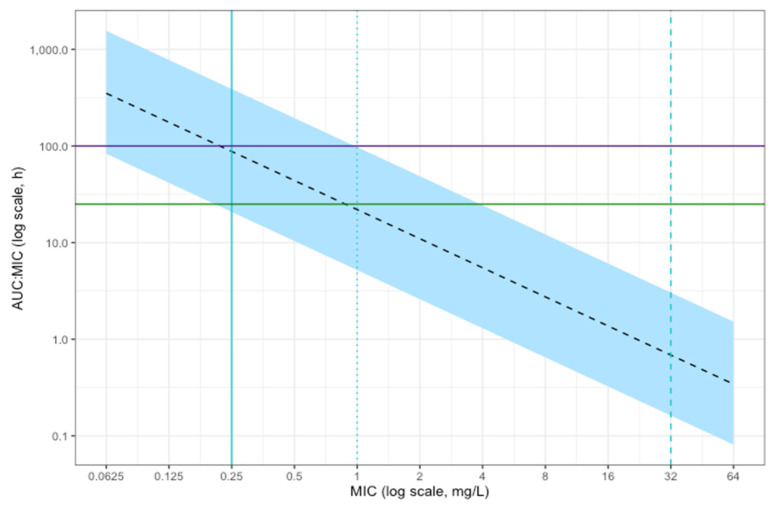
Simulated free 24 h AUC:MIC at steady state and given MIC. The shaded area represents the 95% confidence interval of simulated patients (*n* = 10,000) who achieve the ratio of exposure over 24 h compared to the mean inhibitory concentration (AUC:MIC) at a particular MIC. The dashed black line shows the median AUC:MIC at each MIC. The horizontal lines represent AUC:MIC targets: the green line shows the standard target of AUC:MIC > 25; the purple line represents a more conservative target of AUC:MIC > 100. The vertical lines represent EUCAST breakpoints: 0.25 mg/L for *Streptococcus pneumoniae*, Streptococcal groups A, B, C and G and *Moraxella catarrhalis*; 1 mg/L for *Staphylococcus aureus* and 32 mg/L as the ECOFF for *Haemophilus influenzae*.

The simulation of the free 24 h AUC:MIC of 10,000 patients receiving a dose of intravenous clarithromycin of 500 mg twice daily is shown in Figure 4. This suggested that the majority of simulated patients would achieve the conservative PKPD target of AUC:MIC above 100 for *Streptococcus pneumoniae*, *Moraxella catarrhalis* and Streptococcal groups A, B, C and G isolates considered sensitive to macrolides. Target attainment is reduced for MIC values approaching the 0.25 mg/L resistant breakpoint for these organisms. All patients achieved the standard target of an AUC:MIC > 25 for MIC values below this resistant breakpoint.

At a higher MIC of 1 mg/L, the clinical breakpoint for *Staphyloccocus* spp. (including *Staphylococcus aureus*), less than 50% of simulated patients achieved the standard therapeutic target of an AUC:MIC > 25. The majority of simulated patients achieved the standard therapeutic target of an AUC:MIC > 25 for *Mycoplasma pneumonia* considered sensitive using CLSI methods (MIC below 0.5 mg/L), but most did not achieve the higher target of an AUC:MIC > 100. For *Haemophilus influenzae*, *Legionella pneumophila* and *Chlamydia pneumoniae*, there are no meaningful clinical breakpoints (the epidemiological cut-off of 32 mg/L is suggested as an alternative by the EUCAST for *Haemophilus influenzae*, but the clinical utility of this is unclear). Therefore, meaningful target attainment could not be estimated for these species.

## 3. Discussion

To our knowledge, this is the first study to describe intravenous clarithromycin pharmacokinetics in critically ill adults. A two-compartment model was found to provide the best fit for the data, supported by model evaluation methods which suggested a robust model fit.

Our median estimates for structural parameters are lower than those previously reported values in the literature. The clearance of 8.2 L/h/70 kg is lower than previously reported estimates from oral models. Traunmüller et al. reported separate parameters for healthy volunteers receiving different dosing regimens of 500 mg twice daily and 250 mg twice daily. The reported clearance for the standard regimen of 500 mg twice daily was 18.7 L/h [6]. Fish and Abraham performed a study of clarithromycin administered via a nasogastric tube in critical illness and found a clearance/bioavailability (CL/F) of 28.3 L/h on Day 1 and 27.5 L/h on Day 4 [9]. Abduljalil et al. reported an apparent clearance of 60 L/h in healthy volunteers (bioavailability not measured/assumed), but this study also reported that autoinhibition reduced clearance to 10% of its initial value (closer to our estimate) [7]. Chu et al. examined clarithromycin pharmacokinetics in healthy volunteers and found a clearance/bioavailability of 46.8 L/h with a single 500 mg dose, reducing to 26.2 L/h by the seventh dose [21].

Abduljalil et al. have previously demonstrated that autoinhibition of clarithromycin metabolism occurred within the first 48 h of administration in a study of healthy volunteers and modelled this using a separate inhibition compartment [7]. We tested autoinhibition but did not find evidence of this effect in this critically ill population. This may be due to inherent differences in the populations studied. As noted, the estimate for clearance in our population is lower than the apparent clearance reported by Abduljalil et al. and the effect of autoinhibition may therefore be less significant. There was also no evidence of non-linear pharmacokinetics in this cohort. The low dose range in this cohort may be a limitation in the study’s ability to detect non-linear elimination kinetics, despite the wide range of patient weights (53–120 kg).

The volume of distribution at steady state in this study (86.3 L/70 kg) is lower than previously reported values [6,7,9,21]. Traunmüller et al. reported a volume of distribution of 126.5 L with no reference to bioavailability and with a dosing regimen of 500 mg twice daily [6]. Fish and Abraham reported a volume of distribution of 176.3 L on Day 1 and 174.4 L on Day 4 [9] and Abduljalil et al. reported a value of 172 L, both without reference to bioavailability [7]. Chu et al. reported a volume of distribution with reference to bioavailability (V/F) of 306 L with a single 500 mg dose in healthy volunteers, reducing to 191 L at dose seven [21].

The findings demonstrate very high interindividual variability in structural parameters, particularly in the volume of the central compartment, with more than a 200-fold difference in parameter estimates between individual estimates from 3.1 to 766.4 L/70 kg. There was also a large range of clearance values between 2.1 L/h/70 kg and 55.7 L/h/70 kg. This high pharmacokinetic variability of antimicrobials in critical illness is in keeping with previous findings [3].

Despite highly variable pharmacokinetics, the probability of target attainment is high for the therapeutic target of a free 24 h AUC:MIC > 25 for MIC values of 0.25 mg/L and below (the resistant breakpoint for the majority of macrolide-susceptible species with known clinical breakpoints). Above a MIC of 0.25 mg/L, the probability of achieving this target decreases, and less than 50% of simulated patients achieve this target at a MIC of 1 mg/L (the resistant breakpoint for *Staphylococcus* spp.). The majority of simulated patients also achieve the higher target of an AUC:MIC > 100 with a MIC below 0.25 mg/L, but this probability reduces between MIC values of 0.25 mg/L and 1 mg/L, and no simulated patients achieve this target at the resistant breakpoint for *Staphylococcus* spp. of 1 mg/L. However, these targets are derived from animal models. The correlation with clinical outcomes and the rationale behind aiming for a higher target is unclear from available sources. Further study into PKPD targets for macrolide use in critical illness may be beneficial.

Importantly, clarithromycin is most commonly used to cover the following “atypical pathogens”: *Legionella pneumophila*, *Mycoplasma pneumoniae* and *Chlamydia pneumoniae.* Of these, *Legionella pneumophila* and *Chlamydia pneumoniae* do not have known clinical breakpoints, and there is a lack of international consensus on antimicrobial susceptibility testing for *Mycoplasma pneumoniae*. Therefore, PKPD target attainment for these organisms cannot be estimated.

Our findings can be compared to the study by Fish and Abraham (1999) [9]. This was a study of clarithromycin pharmacokinetics when administered via a nasogastric tube in critically ill patients [9]. APACHE II scores for disease severity are comparable between the two studies: Fish et al. studied a population with a median APACHE II of 19 and a range of 14 to 24, while our sample of patients had a median APACHE II of 20 and a range of 0 to 28. However, this study examined oral clarithromycin in patients who were suitable for an intravenous to oral switch and had no evidence of renal, hepatic or gastrointestinal dysfunction. In comparison, the majority of our participants were receiving vasopressor support during the study and there was significant variation in renal function and evidence of hepatic dysfunction. Therefore, the patients studied by Fish et al. could be considered to have a very different phenotype of critical illness compared to our population. Fish et al. found limited intrapatient and interpatient variability of clarithromycin pharmacokinetics. In comparison, our findings show substantial variability between structural pharmacokinetic parameters for individuals. Fish et al. found no significant difference in secondary pharmacokinetic parameters between Day 1 and Day 4 of their study. Similarly, we found no evidence of interoccasional variability in our study.

This study has a number of limitations. There was a relatively high uncertainty of parameter estimates in our model and a larger dataset may be more informative. We did not measure the metabolite 14-OH-clarithromycin, which possesses antimicrobial activity. However, Abduljalil et al. (2019) have previously noted that AUC of clarithromycin is approximately three times as high as its metabolite; the majority of the antibiotic activity is likely to be derived from clarithromycin [7]. As clarithromycin exposure significantly exceeded the target range in many patients, clarithromycin toxicity may have been a concern, which this study did not assess or model. This study did not assess pharmacodynamic data, such as clinical outcomes, which would be necessary to clarify the PKPD target for clarithromycin in critical illness for treatment success. In addition, we did not assess the immunomodulatory effects of clarithromycin, which may have an additive effect on clinical outcomes.

Our study suggests that, despite high interindividual pharmacokinetic variability, PKPD target attainment for clarithromycin in critically ill patients is reasonable for most target organisms with known clinical breakpoints. When treating organisms with a higher MIC, even if considered sensitive with known breakpoints, higher PKPD targets may not be achieved. Although the clinical breakpoints for many important pathogens that clarithromycin is commonly used to cover are not known, this investigation does illustrate the pharmacokinetic profile expected in critically ill patients. The clinical utility for these “atypical” pathogens will emerge as understanding of these organisms develops.

Further clarity over clinical breakpoints for relevant organisms, PKPD targets and correlations with treatment success is needed to define optimal clarithromycin dosing.

## 4. Materials and Methods

Participants were enrolled in the ABDose study. The methods for this study have been previously described in detail [22,23]. Adults admitted to the critical care unit of St. George’s Hospital in London, United Kingdom, who received intravenous clarithromycin were recruited. Exclusion criteria included previous enrolment in the ABDose study, treatment withdrawal for palliation or expected prognosis of less than 48 h from enrolment. Informed consent, or next of kin assent in cases of temporary incapacity due to critical illness, was obtained. In cases of assent, informed consent was obtained once participants regained capacity. Ethical approval was given by the National Research Ethics Committee London (REC reference 14/LO/1999). The study was sponsored by St George’s University of London (Joint Research Office reference 14.0195). The study was conducted in accordance with the Declaration of Helsinki.

Data were collected from clinical notes, including baseline demographic data and clinical information. Drug administration data were collected from electronic prescriptions and infusion pumps to ensure accuracy in administration times. Clarithromycin is usually given as an infusion over 1–2 h in local clinical practice. Sampling was based on the indicative schedule in Table 3, but a pragmatic and opportunistic strategy was employed, timing samples with clinical collection as far as possible. A maximum of eight samples were taken from any participant. Blood samples were taken from radial arterial lines. The samples were placed immediately upon ice and plasma was separated using centrifugation. Processed samples were stored at −80 °C. Samples were analysed in batches and total clarithromycin concentrations were measured by Analytical Services International Ltd. Ultra-high-performance liquid chromatography–tandem mass spectrometry equipped with a Waters TQ detector (Waters, Milford, MA, USA) was performed using Waters MassLynx software version 4.1 (Waters, Milford, MA, USA). This method has previously been described [24].

Population pharmacokinetic analysis was performed using a non-linear mixed effects approach implemented in NONMEM^®^ modelling software (version 7.5.1, Dublin, Ireland) [25] operating with GForTran via Homebrew (version 13.2.0). Weight was added a priori with an allometric exponent of 0.75 for clearance parameters and 1 for compartment volumes, as previously described [26]. Interindividual variability was modelled assuming log-normally distributed parameters. Periods during which time participants were receiving renal replacement therapy were excluded from the analysis. One-, two- and three-compartment models were tested, followed by covariate analysis and the addition of interoccasion variability.

Models were evaluated and selected using a combination of biological plausibility, numerical and visualisation methods. Numerical methods included minimisation of the NONMEM objective function (OFV). This required nested models with one additional parameter to show a minimum reduction of 3.84 for a significant improvement in model fit for a significance level of *p* < 0.05. Diagnostic plots were produced using the R packages xpose4 (version 4.7.3) [27] and Perl-speaks NONMEM (version 5.3.1) [28] with R version 4.2.3. Simulations of 10,000 patients receiving intravenous clarithromycin at the World Health Organization’s [29] and Infectious Diseases Society of America’s [30] recommended dose of 500 mg every 12 hours were performed based on the final pharmacokinetic model and using estimated protein binding of 80% [13]. Target attainment for clinical targets of a free 24 h AUC:MIC at steady state of 25–35 [14] or over 100 [15] was predicted. The R code for simulations is available in the Supplementary Material.

## Figures and Tables

**Table 1:** Summary of patient characteristics.

Characteristic	Median or Total	Range (Interquartile Range)
Age (years)	66	25–85.8 (56.7–72.7)
Weight (kg)	80	53–120 (65–95)
Height (cm) *	173	150–192 (166–178)
BMI (kg/m^2^) *	26.9	18.3–35.2 (22.9–31.0)
Creatinine (µmol/L)	89	40–276 (71–121)
Albumin (g/L)	25	12–38 (21–29)
APACHE II score	20	0–28 (16–23)
CRP (mg/L)	70	3.5–478 (50.3–169.8)
ALT (U/L)	34	9–166 (24–48)
Male:Female	12:7	
Ethnicity		
White	12	
Black British	1	
Asian	1	
Not stated	5	
Infection source **		
Chest	14	
Skin	1	
Central nervous system	1	
Ear nose and throat	2	
Gastrointestinal	1	
Unknown	1	
Vasopressors (no. of patients)	12	
Ventilation status **		
Self-ventilating	14	
Non-invasive ventilation	4	
Invasive ventilation	12	
Clinical outcome (90 days)		
Alive	13	
Deceased (infection-attributable)	3	
Deceased (not attributable to infection)	3	

* One participant did not have a height measurement recorded. ** Participants may have more than one source or ventilation status during the study period.

**Table 2:** Parameter estimates.

	Mean Parameter Estimates	Relative Standard Error (%)	Range of Individual Estimates	Bootstrap (*n* = 500) Median (95% CI)
Fixed effects				
*θ*_CL (L/h/70 kg)_	8.17	17	2.1–55.7	7.8 (5.6–10.7)
*θ*_V1 (L/70 kg)_	25.7	29	3.1–766.4	25.3 (5.4–45.0)
*θ*_Q (L/h/70 kg)_	62.0	18		62.4 (43.4–112.5)
*θ*_V2 (L/h/70 kg)_	60.6	16	45.9–112.5	62.0 (44.7–108.0)
OMEGA				
*η*^2^_CL_	0.53	32		0.51 (0.21–0.87)
*η*^2^_V1_	1.55	70		1.58 (0.15–7.95)
SIGMA				
*σ*^2^_Proportional_	0.034	30		0.0317 (0.0161–0.0553)

Half life: 7.8 h.

**Table 3:** Sampling schedule.

	Dosing Interval 1	Dosing Interval 2
Hours after dose	0.5/1, 2, 6, 11.75	0.5/1, 8, 10, 11.75

## Data Availability

The raw data are unfortunately unavailable as permission for open publication of pseudoanonymised data was not sought at time of consent of participants.

## Supplementary Materials

The following supporting information can be downloaded at: https://www.mdpi.com/article/10.3390/antibiotics14060559/s1, Supplementary Code 1: Simulation code.r; Supplementary Material: supplementary_dataS1_concomitant_medication.
